# Unc-51-like kinase 1 (ULK1) regulates bacterial ubiquitylation and p62 recruitment during xenophagic clearance of *Listeria monocyt*ogenes

**DOI:** 10.1128/msphere.00308-25

**Published:** 2025-08-25

**Authors:** J. S. Ribeiro, M. S. Siqueira, T. S. M. Farias, T. D. P. Spinasse, P. L. O. Correia, R. M. Ribeiro, F. M. Bastos de Oliveira, L. A. M. Carneiro, L. H. Travassos

**Affiliations:** 1 Laboratory of Immunoreceptors and Signaling, Institute of Biophysics Carlos Chagas Filho, Federal University of Rio de Janeiro 204227, Rio de Janeiro, Brazil; 2 Laboratory of Innate Immunity and Inflammation, Department of Immunology, Institute of Microbiology Paulo de Góes, Federal University of Rio de Janeiro 341406, Rio de Janeiro, Brazil; University of Kentucky College of Medicine, Lexington, Kentucky, USA

**Keywords:** xenophagy, autophagy, ULK1, p62, ubiquitination, *Listeria monocytogenes*, infection, bacterial clearance, innate immunity, host-microbe interactions

## Abstract

Autophagy is an essential cellular homeostatic process that also serves as an innate immune mechanism against intracellular bacterial pathogens through a highly selective form of autophagy known as xenophagy. Despite advances in understanding how bacteria are targeted for autophagic degradation, the specific regulatory mechanisms that drive the initial steps and ensure bacterial selection remain incompletely defined. Our study uncovers a pivotal role for Unc-51-like kinase 1 (ULK1) in the xenophagic clearance of the intracellular bacterial pathogen *Listeria monocytogenes*. We observed that ULK1 is essential for the efficient ubiquitylation of bacteria and subsequent recruitment of the autophagic adaptor protein p62 to the bacterial surface. Furthermore, we show that the impact of ULK1 deficiency in these early events—reduction in bacterial ubiquitylation, followed by impaired p62 targeting, results in diminished formation of bacteria-targeted autophagosomes. Notably, phosphorylation of p62 at the S409 residue, which is known to be dependent on ULK1 to enhance its affinity for ubiquitin, is necessary for the recruitment of p62 to the bacterial surface and adequate bacterial clearance, highlighting the regulatory role of ULK1 in this process. These findings unveil a previously unrecognized function of ULK1 in modulating early xenophagy steps, contributing to the autophagic control of intracellular pathogens. Our findings offer new perspectives into the manipulation of ULK1 activity for therapeutic interventions against infectious diseases.

**IMPORTANCE:**

Autophagy is a vital process in eukaryotic cells that enables them to digest intracellular components, helping them respond to various stresses, including starvation, the accumulation of dysfunctional organelles, and infections. While the autophagic flux has been extensively studied over the past few decades, some key mechanisms remain poorly understood. Our research aimed to clarify one such mechanism: how the autophagic machinery specifically targets intracellular bacteria. We identified a novel role for the protein ULK1 in this process, demonstrating that ULK1 is essential for tagging bacteria with ubiquitin within the cell and recruiting the protein p62. These are critical steps for adequate bacterial clearance. Our results underscore the pivotal role of ULK1 in initiating the cellular defense against bacterial infections. Our findings could pave the way for new therapeutic strategies to enhance the body’s capacity to combat bacterial infections.

## INTRODUCTION

Autophagy is a cellular process highly conserved in eukaryotes that maintains homeostasis at cellular and systemic levels. During its activation, portions of the cytosol are sequestered into double-membrane vesicles called autophagosomes that can either fuse with late endosomes to form amphisomes or directly with lysosomes, resulting in the degradation of their content (1). Although initially thought of as a non-selective bulk degradation process responsible for maintaining energy levels during nutrient deprivation, in the last two decades, evidence accumulated that autophagy can be a highly selective process in which a variety of adaptor proteins recruit the autophagy machinery to specific cargos (2).

Studies from the early 2000s demonstrated that autophagy could target several intracellular bacterial pathogens, such as group A *Streptococcus* (3), *Mycobacterium tuberculosis* (4), and *Shigella flexneri* (5), in a specific form of autophagy called xenophagy. These studies were instrumental in establishing xenophagy as a vital effector mechanism of the innate immune defense arsenal.

Recognition of bacteria or bacterial products by surface (6) or cytosolic pattern recognition receptors (7, 8) has been implicated in the initiation of xenophagy. Still, they alone cannot explain how the formation of new autophagosomes is directed to specifically engulf bacteria in the cytosol. Much effort has been put into understanding the initial steps of xenophagy, mainly how bacteria are targeted as cargo for autophagy. Currently, it is known that the activity of different ubiquitin ligases, such as leucine-rich repeat and sterile-motif-containing 1 (9), Parkin (10), SMAD-specific E3 ubiquitin protein ligase 1 (Smurf1) (11), and Nedd4-1 (12) results in the decoration of intracellular bacteria with ubiquitin. This is a crucial event to target cytosolic bacteria to autophagosomes because it allows autophagic adaptors, such as p62 (13), nuclear dot protein 52 kDa (NDP52) (14), and optineurin (15), which harbor both ubiquitin-binding domains and LC3-interacting region motifs in their structure, to act as bridges between the ubiquitylated bacteria and phagophores (16). Despite all these advances in the knowledge regarding the involvement of ubiquitin ligases and autophagic adaptors, much is to be learned concerning the players that regulate this critical step for xenophagy.

Unc-51-like kinase 1 (ULK1) is a serine-threonine kinase that is part of the ULK1 complex, consisting of ULK1, the non-catalytic focal adhesion kinase family interacting protein of 200 kDa (FIP200), ATG13, and ATG101 (17). This core autophagy initiation complex is present on phagophore membranes and, in concert with the class III phosphatidylinositol 3-kinase complex (Beclin 1, ATG14L, Vps34, and Vps15), regulates the early steps of autophagosome formation (17, 18). Phagophore expansion into an autophagosome requires additional proteins such as WD-repeat protein Interacting with PhosphoInositides, ATG9, ATG2, and two ubiquitin-like systems: the ATG12-ATG5 system, in which ATG12 is conjugated to ATG5 to form a complex consisting of ATG12, ATG5, and ATG16L1. This complex is necessary for the conjugation of phosphatidyl-ethanolamine to the ATG8 system, a family of proteins present on the membrane of the nascent autophagosome (19).

Previous studies demonstrate that the ULK1 complex detects upstream signals from energy-sensing pathways, such as mammalian Target of Rapamycin (mTOR) and AMP-activated protein kinase (AMPK), thus playing a central role in regulating intracellular energy levels (17). mTOR and AMPK are master regulators of nutrient/energy levels and have been demonstrated to regulate ULK1 activity through phosphorylation at different sites (20–23). Although the essential role of ULK1 in the initiation of starvation-induced autophagy is undisputed, its participation in bacteria-targeted xenophagy is far from being elucidated. Here, we investigated the role of ULK1 in xenophagy, specifically in how it participates in the initial events of bacterial ubiquitylation and p62 recruitment to the bacterial surface that will result in bacterial clearance by autophagy. For this purpose, we used *Listeria monocytogenes,* an intracellular bacterial pathogen that escapes from phagosomes to replicate in the cytosol of host cells. This bacterium represents an excellent experimental model as it has long been reported to trigger ubiquitylation and p62 recruitment to its surface, leading to the formation of autophagosomes around bacteria (24). We demonstrate that infection of mouse embryonic fibroblasts (MEFs) with *L. monocytogenes* leads to the recruitment of ubiquitin and p62 to the bacterial surface, forming LC3^+^ compartments colocalizing with bacteria. Moreover, we found that this process depends on the escape to the cytosol as phagosome-confined *L. monocytogenes* do not recruit xenophagy markers nor colocalize to autophagosomes. More importantly, we show that, in ULK1-KO MEFs, bacteria are not efficiently ubiquitylated, and p62 is not targeted to the bacterial surface throughout infection with *L. monocytogenes*. In addition, we demonstrate that the phosphorylation of p62 at the S409 residue, known to be dependent on ULK1 (25) and to increase its affinity to ubiquitin, is required to recruit p62 to the bacterial surface. Finally, we demonstrate that ULK1 deletion or the S409A substitution that impairs p62 phosphorylation resulted in significantly diminished targeting of bacteria to autophagosomes and increased numbers of intracellular bacteria. Taken together, our results point to an unrecognized role of ULK1 in controlling bacterial ubiquitylation and p62 recruitment to the surface of cytosolic bacteria, which contributes to bacterial control.

## RESULTS

### Cytosolic *L. monocytogenes* triggers xenophagy

**Fig 1:**
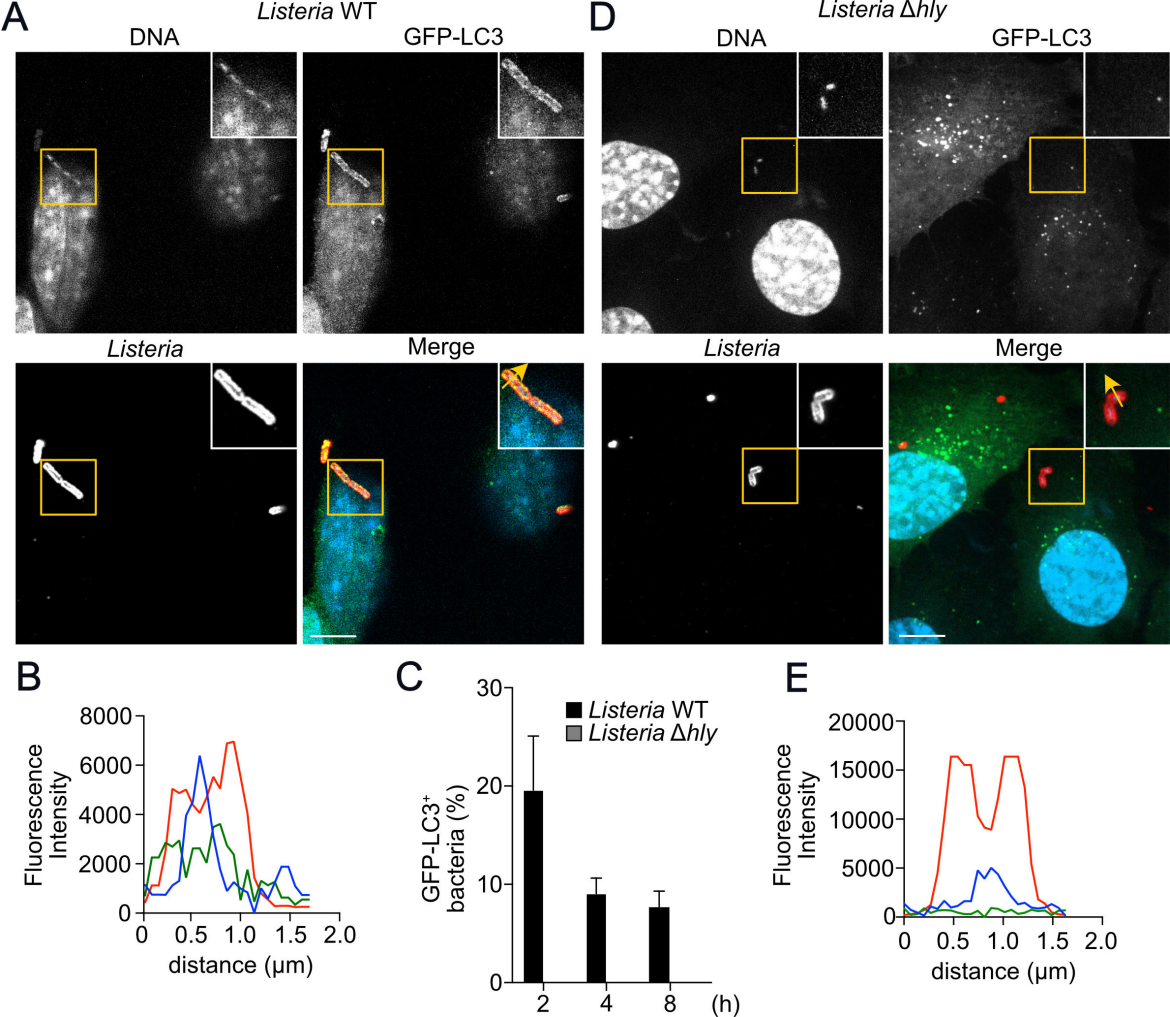
*L. monocytogenes*-targeted autophagy depends on listeriolysin O (LLO) expression. (**A**) Representative confocal images of MEFs GFP-LC3 infected with wild-type or Δ*hly L. monocytogenes* for 2 h and stained for *Listeria* (red) and DNA (blue). (**B**) Plot profiles of fluorescence intensity along the yellow arrow traced in the insets in (**A**), GFP-LC3 (green line), *Listeria* (red line), and DNA (blue line). (**C**) Quantification of the number of intracellular wild-type and Δ*hly L. monocytogenes* colocalizing with GFP-LC3 at 2, 4, and 8 h. (**D**) Representative confocal images of MEFs GFP-LC3 infected with Δ*hly L. monocytogenes* and stained for *Listeria* (red) and DNA (blue). (**E**) Plot profiles of fluorescence intensity along the yellow arrow traced in the insets in (**D**); GFP-LC3 (green line), *Listeria* (red line), and DNA (blue line). Values are means ± SEM. Scale bars are 10 µM, *N* = 3.

It has been described that *L. monocytogenes* and other intracellular bacteria interact with the autophagic machinery in multiple ways (24–26). These bacteria have evolved mechanisms that allow them to manipulate this host response and, in some cases, even take advantage of the autophagy response. To quantify *L. monocytogenes* targeting and kinetics of autophagy response in our model, we infected wild-type MEFs transduced with GFP-LC3 to evaluate the recruitment of the autophagy marker LC3 to the bacterial surface. Immunofluorescence experiments demonstrated the association of LC3 with the wild-type strain of *L. monocytogenes*, indicating that xenophagy was triggered at 2 h (Fig. 1A and B). To precisely determine the dynamics of LC3 recruitment to the bacterial surface, MEFs were infected for 1, 2, 4, or 8 h, and the number of cytosolic bacteria associated with LC3 was determined by immunofluorescence. As shown in Fig. 1C *L*. *monocytogenes* association with LC3 was maximal at 2 h post-infection (~20% of total bacteria). Xenophagy can target different intracellular bacterial species that survive in the cytosol (7) or damaged vacuoles (27). To determine in which intracellular compartment *L. monocytogenes* was targeted by xenophagy, we used a deletion mutant of *L. monocytogenes* that lacks listeriolysin O (LLO), a cholesterol-dependent pore-forming cytolysin (encoded by the *hly* gene) that is unable to escape from phagosomes and access the host cytosol, as previously demonstrated (28–30). We confirmed that bacteria retained in intact vacuoles do not initiate autophagy, as Δ*hly L. monocytogenes* did not associate with LC3 (Fig. 1C through E). It has been demonstrated that *L. monocytogenes* infecting macrophages can establish replicative niches termed SLAPs (Spacious Listeria-containing phagosomes) (31). More recently, similar compartments called eSLAPs (epithelial SLAP-like) were described in epithelial cells (32). Both SLAPs and eSLAPs are non-acidic and non-degradative vacuoles characterized by the recruitment of LC3 and require the secretion of LLO. To exclude the possibility that the recruitment of LC3 to the surface of *L. monocytogenes* observed in Fig. 1A was due to the formation of eSLAPs, we infected GFP-LC3 MEFs with the wild-type strain of *L. monocytogenes* for 1 h and stained for p62, as SLAPs are not decorated with this protein or with ubiquitin. As demonstrated in Fig. S1A nad B, GFP-LC3-positive bacteria are also positive for p62. Similar results were obtained with the staining of endogenous LC3 (Fig. S1C and D). These results confirm that the association of bacteria with LC3 described in Fig. 1A through C represents autophagosomes rather than eSLAPs.

### Ubiquitin and p62 are recruited to *L. monocytogenes* surface

**Fig 2:**
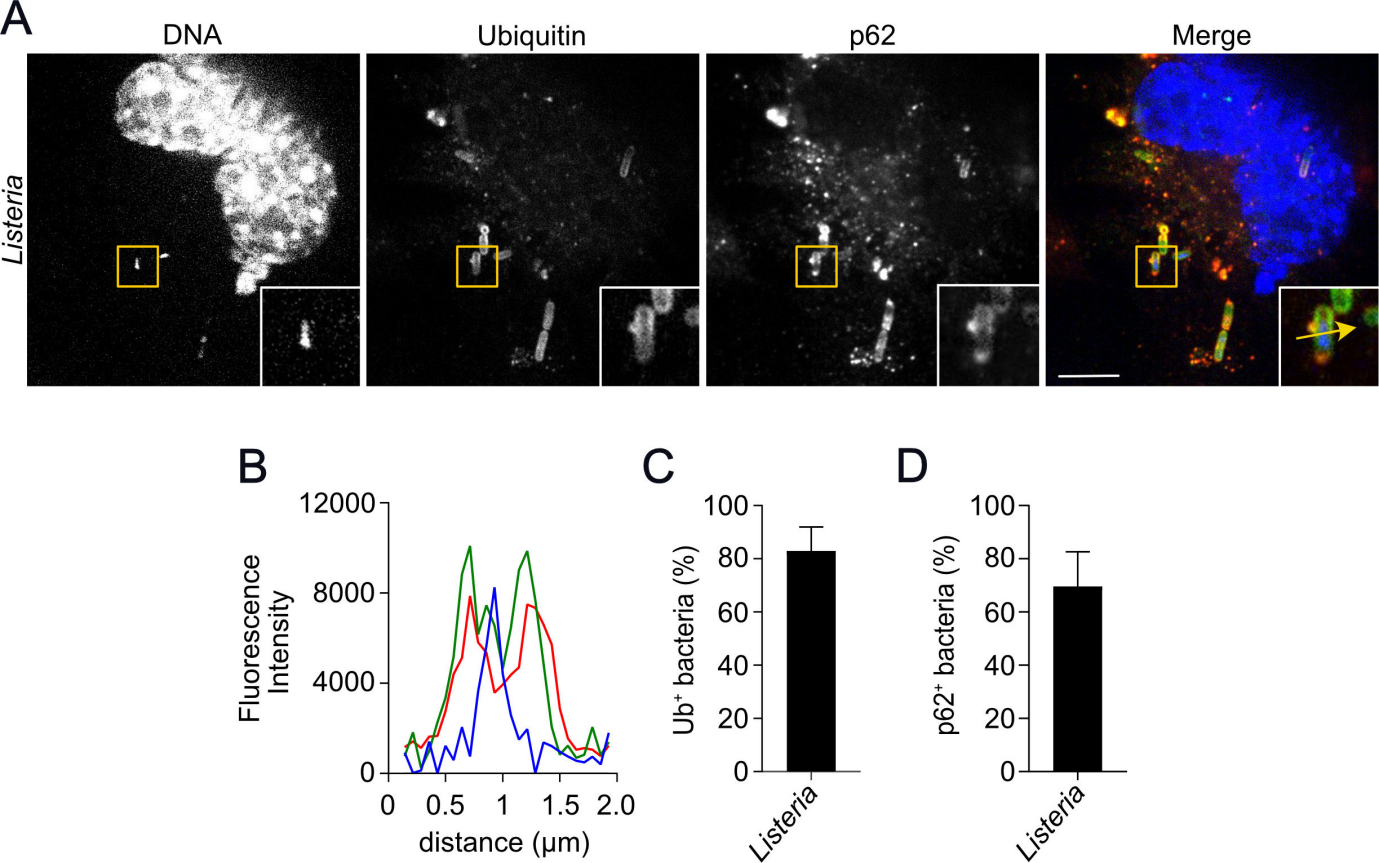
p62 is recruited to the surface of *L. monocytogenes* early during infection. (**A**) Representative confocal images of MEFs infected with wild-type *L. monocytogenes* for 2 h and stained for ubiquitin (green), p62 (red), and DNA (blue). (**B**) Plot profiles of fluorescence intensity along the yellow arrow traced in the inset in (**A**); ubiquitin (green line), *Listeria* (red line), and DNA (blue line). (**C**) and (**D**) Quantification of the number of ubiquitin-positive (Ub^+^) (**C**) and p62-positive (p62^+^) (**D**) *L. monocytogenes*. Values are mean ± SEM. Scale bars are 10 µM, *N* = 3.

Since the description of xenophagy as a cell-autonomous mechanism that deals with intracellular bacteria, it has become clear that early events of bacterial ubiquitylation are essential to allow the recruitment of autophagy adaptors that will direct the formation of the autophagosomes around bacteria. To understand these crucial events for *L. monocytogenes*-induced autophagy, we started by analyzing the recruitment of ubiquitin during the infection. To this end, wild-type MEFs infected with either wild-type or Δ*hly L. monocytogenes* for 2 h and stained for ubiquitin , and the number of ubiquitin-positive bacteria was quantified. Consistent with the findings described above, the LLO-deleted strain of *L. monocytogenes* was not stained with Ub, in sharp contrast to what was observed with the wild-type strain, which had ~50% of the intracellular bacterial population Ub^+^ as early as 2 h post-infection (Fig. S2A through C). Once ubiquitylated, bacteria may be targeted to autophagy by several ubiquitin-binding adaptors. Among them, p62 emerges as the best-characterized one and has been consistently reported to bind ubiquitin-coated bacteria in the cytosol (33). Thus, we analyzed the recruitment of p62 to the surface of Ub-positive *L. monocytogenes*. For this purpose, we immunostained wild-type MEFs infected with wild-type *L. monocytogenes* for Ub and p62. Our analysis demonstrates that most of the bacterial population (~65%) was Ub and p62^+^ positive, in line with the notion that p62 is implicated in detecting ubiquitylated cytosolic bacteria (Fig. 2A through D).

### ULK1 is essential for ubiquitylation and recruitment of p62 to *L. monocytogenes* surface

**Fig 3:**
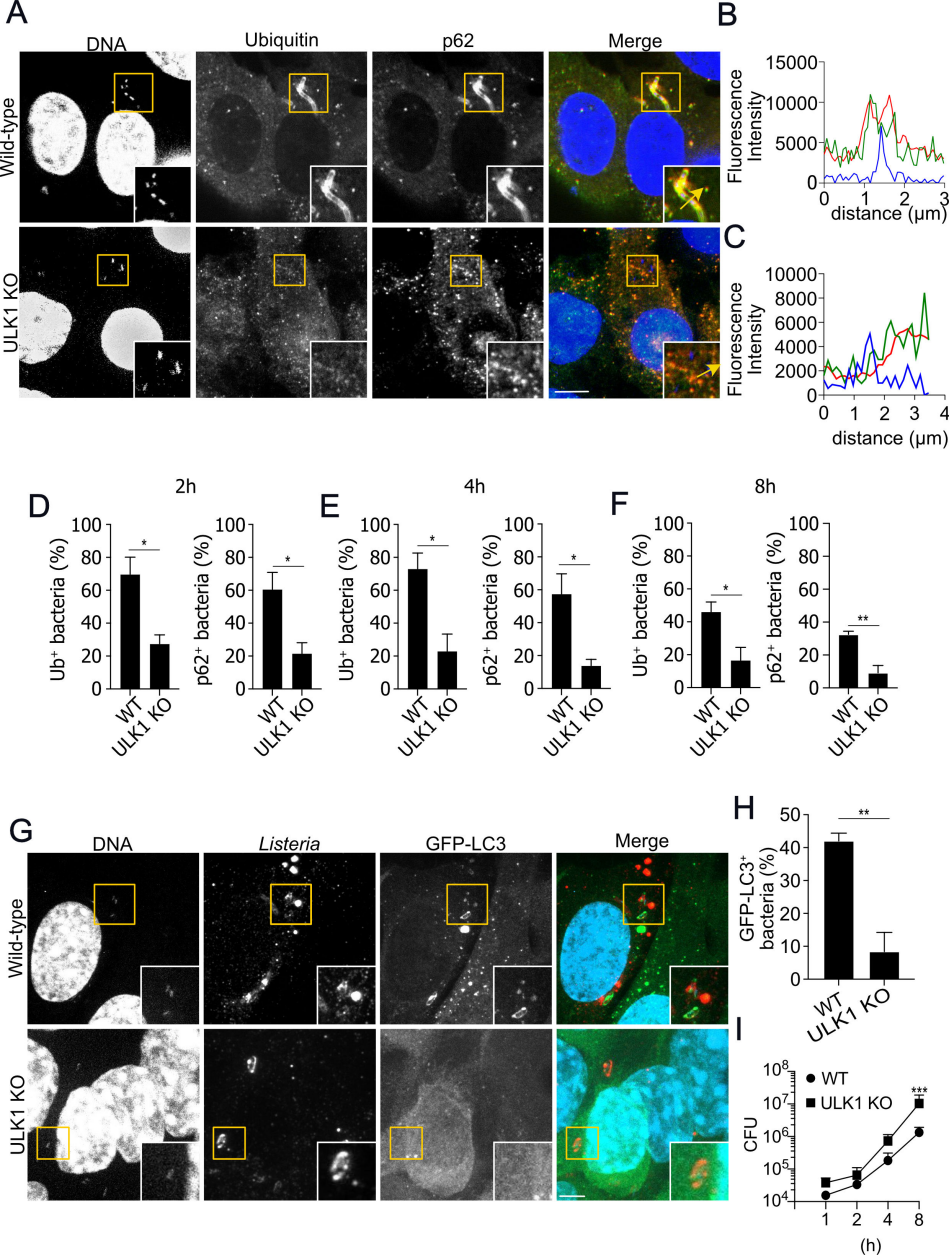
ULK1 is required to efficiently recruit ubiquitin and p62 and restrict the intracellular growth of *L. monocytogenes*. (**A**) Representative confocal images of wild-type (WT) and ULK1 knockout (ULK1 KO) MEFs infected with *L. monocytogenes* for 2 h and stained for ubiquitin (green), p62 (red), and DNA (blue). (**B**) and (**C**) Plot profiles of fluorescence intensity along the yellow arrow traced in the insets in (**A**); ubiquitin (green line), *Listeria* (red line), and DNA (blue line) in WT (**B**) and ULK1 KO MEFs (**C**) infected with *L. monocytogenes*. (**D–F**) Quantification of the number of Ub^+^ and p62^+^ *L. monocytogenes* in WT and ULK1 KO MEFs infected for 2 h (**D**), 4 h (**E**), and 8 h (**F**), *P < 0.05 and **P < 0.01, Unpaired *t*-test, *N* = 5. (**G**) Representative confocal images of WT and ULK1 KO MEFs expressing GFP-LC3 and infected with *L. monocytogenes* for 2 h, GFP-LC3 (green), *Listeria* (red), and DNA (blue). Scale bars are 10 µM. Values are mean ± SEM. (**H**) Quantification of the number of *L. monocytogenes* positive for GFP-LC3 in WT and ULK1-KO MEFs infected for 2 h, **P < 0.01, Unpaired *t*-test. (**I**) Intracellular growth curves of WT and ULK1-KO MEFs infected with *L. monocytogenes*. ***P < 0.001, one-way ANOVA, *N* = 3.

Compelling evidence from the literature establishes that infection of epithelial cells with intracellular bacterial pathogens such as *S. flexneri* (34) and *L. monocytogenes* (35) induces a state of amino acid starvation, as determined by the early delocalization of mTOR from lysosomes. We performed immunofluorescence analysis of mTOR and Lamp1 distribution in wild-type MEFs infected with *L. monocytogenes* and observed a substantial reduction in the area of colocalization of mTOR and Lamp1 throughout infection, as previously demonstrated (35) (Fig. S3A through C)). Based on previous data from the literature, our findings suggest that *L. monocytogenes* induces early amino acid starvation in our infection model, an event known to activate ULK1 to initiate autophagosome formation (20, 21), which led us to hypothesize whether ULK1 could also play a role in the control of ubiquitylation and recruitment of p62 to *L. monocytogenes* surface. To test this, we infected wild-type and ULK1 knockout MEFs (ULK1 KO) with wild-type *L. monocytogenes* and analyzed the recruitment of these two proteins by immunofluorescence. As shown in Fig. 3A, B and D at 2 h post-infection, ~70% of the bacterial population in the cytosol of wild-type MEFs was coated with ubiquitin and ~60% with p62. In contrast, we observed that only ~27% and ~31% of *L. monocytogenes* in the cytosol of ULK1 KO MEFs were decorated with Ub and p62, respectively (Fig. 3A, C and D). Proteins related to autophagosome formation, such as ATG5, have been linked to the escape from the phagocytic vacuoles in MEFs (36). To investigate whether the reduction in the recruitment of ubiquitin and p62 to the bacterial surface in ULK1 KO MEFs was due to an escape defect, the ability of *L. monocytogenes* to escape from primary vacuoles was examined in both wild-type and ULK1 KO MEFs infected with wild-type *L. monocytogenes* and stained with phalloidin for the quantification of phalloidin-positive (escaped) bacteria. Our results show that wild-type *L. monocytogenes* escaped at equal rates from primary vacuoles in wild-type and ULK1 KO MEFs (Fig. S4). Finally, to exclude the possibility that the reduction in bacterial ubiquitylation and p62 recruitment in ULK1 KO MEFs was due to delayed kinetics, we infected wild-type and ULK1 KO MEFs for 4 and 8 h and observed similar results. At 4 h post-infection, we found that ~73% versus~22% of bacteria were positive for ubiquitin and ~57% versus ~13% positive for p62 in wild-type versus ULK1 KO MEFs, respectively (Fig. 3E; Fig. S5A). Similar results were observed at 8 h post-infection (Fig. 3F; Fig. S5B).

### ULK1 is not required for the ubiquitylation and recruitment of p62 to *L. monocytogenes* lacking ActA

Upon infection, *L. monocytogenes* mediates its escape from the entry vacuole by secreting LLO and two phospholipases C (PlcA and PlcB) (37, 38). Once free in the cytosol, *L. monocytogenes* masks its surface with ActA, a bacterial protein that promotes host actin polymerization, which the bacteria use for their intracellular motility (39). Interestingly, this ActA mask has been shown to avoid Ub accumulation and p62 recruitment to bacterial surfaces (40, 41). Therefore, we aimed to understand if ActA expression would affect the ULK1-dependent ubiquitylation and p62 recruitment to the *L. monocytogenes* surface. We used a Δ*actA* mutant to infect wild-type MEFs for 2, 4, and 8 h to monitor Ub and p62 coating of bacteria by immunofluorescence. The analysis of the colocalization of Ub and p62 with Δ*actA L. monocytogenes* revealed that virtually all bacteria were densely coated by both proteins throughout the infection (Fig. S6A through D), confirming previous findings showing that the lack of ActA favors bacterial coating with Ub and p62. Next, we infected wild-type and ULK1 KO MEFs with the Δ*actA L. monocytogenes* mutant and analyzed the recruitment of Ub and p62. Immunofluorescence and plot profile analysis demonstrate that ULK1 deficiency does not impact the ubiquitylation and p62 recruitment to the surface of Δ*actA L. monocytogenes* (Fig. S7A through C). To confirm these findings, we quantified the number of Δ*actA* bacteria associated with Ub and p62. In sharp contrast to the results with wild-type bacteria (Fig. 3), ULK1 was not required for the ubiquitylation of Δ*actA L. monocytogenes* and p62 recruitment, as demonstrated by the similar recruitment of Ub and p62 in wild-type and ULK1 KO MEFs infected with Δ*actA L. monocytogenes* (Fig. S7D through F and Fig. S8A and B). Together with the results from previous sections, our data demonstrate that ULK1 plays a crucial role in the early steps of xenophagy by controlling the ubiquitylation and recruitment of p62 to the surface of *L. monocytogenes* expressing ActA.

Next, we investigated whether the defect in ubiquitylation and p62 recruitment observed in ULK1 KO MEFs impacts the targeting of wild-type *L. monocytogenes* to autophagosomes. For this purpose, wild-type and ULK1 KO MEFs expressing GFP-LC3 were infected with wild-type *L. monocytogenes* for 2 h to quantify GFP-LC3-positive bacteria. We show that ULK1 KO MEFs displayed reduced numbers of bacteria associated with GFP-LC3 (Fig. 3G and H), in line with the notion that ubiquitin and p62 recruitment to the surface of cytosolic bacteria are key steps in the targeting bacteria to autophagosomes. Finally, to analyze whether the decrease in the number of *L. monocytogenes*-targeted autophagosomes impacts the viability of bacteria, we performed gentamicin protection assays for the quantification of viable bacteria in wild-type and ULK1 KO MEFs infected with *L. monocytogenes*. Our data demonstrate that ULK1 KO MEFs harbor increased numbers of viable bacteria compared to wild-type MEFs (Fig. 3I). Altogether, these results imply that ULK1 is essential for the ubiquitylation and recruitment of p62 to the surface of *L. monocytogenes* and that its deficiency leads to defective formation of bacteria-targeted autophagosomes and increased intracellular replication.

### Phosphorylation at p62 S409 is required for the recruitment of p62

**Fig 4:**
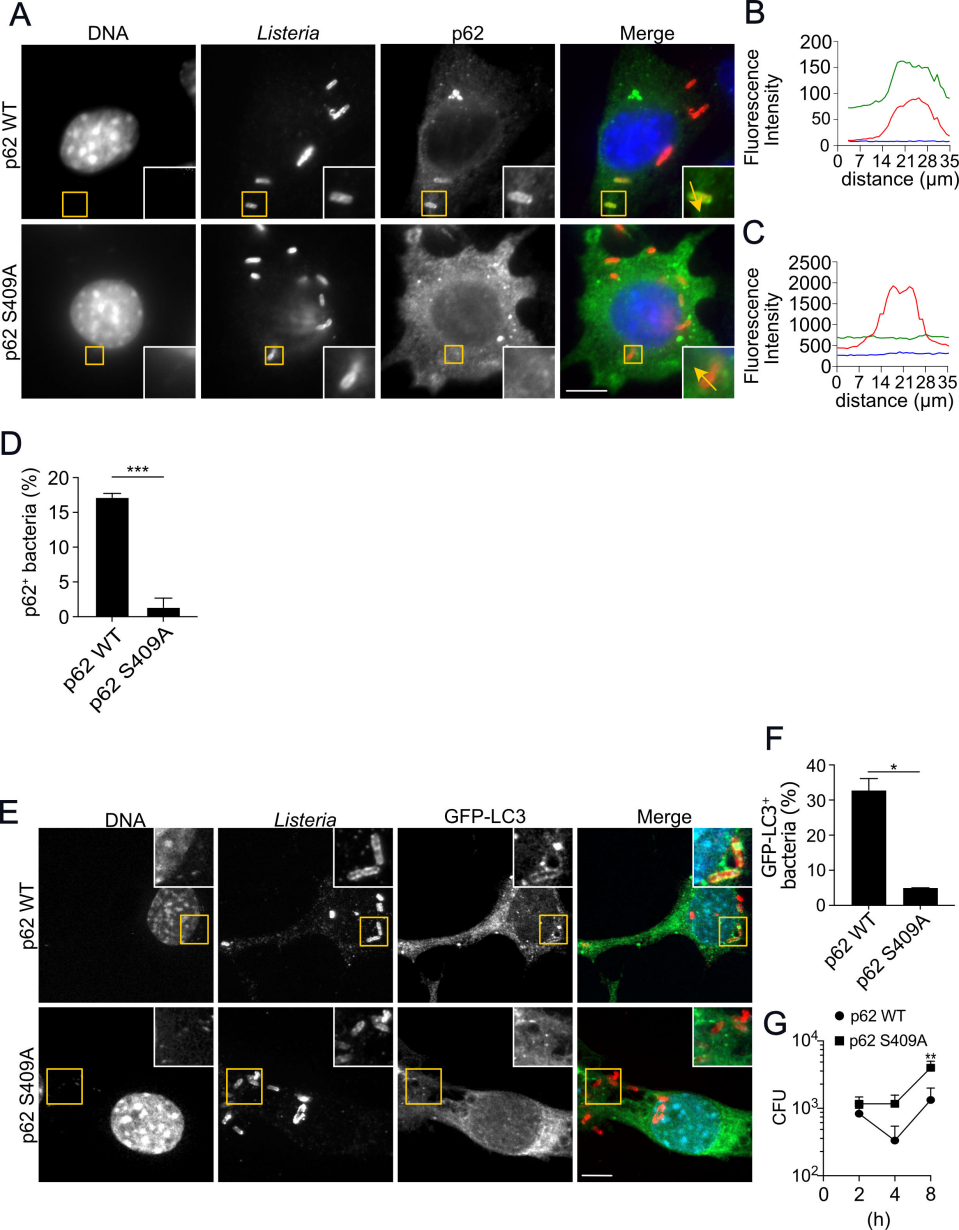
Phosphorylation at p62 S409 is essential for p62 recruitment and *L. monocytogenes* in the cytosol. (**A**) Representative images of p62 knockout MEFs transduced with a retrovirus for the stable expression of FLAG-p62 wild-type (p62 WT) or FLAG-p62 S409A (p62 S409A) infected with *L. monocytogenes* for 2 h and stained for *Listeria* (red), FLAG (green), and DNA (blue). (**B**) and (**C**) Plot profiles of fluorescence intensity along the yellow arrow traced in the inset in (**A**); (**B**) MEFs -p62 WT and (**C**) p62 S409A. FLAG (green line), *Listeria* (red line), and DNA (blue line). (**D**) Quantification of *L. monocytogenes* positive for p62 (p62^+^) in p62 WT and p62 S409A, infected as described in (**A**), ***P < 0.001, Unpaired *t*-test. (**E**) Representative confocal images of p62-KO MEFs stably expressing FLAG-p62 WT or FLAG-p62 S409A transduced with GFP-LC3, infected as in (**A**). (**F**) Quantification of the number of p62-positive bacteria in p62 WT or p62 S409A MEFs, *P < 0.05, Unpaired *t*-test. (**G**) Intracellular growth curves of MEFs stably expressing FLAG-p62 WT or FLAG-p62 S409A infected with *L. monocytogenes*, **P < 0.01, one-way ANOVA. Scale bars are 10 µM. Values are mean ± SEM. *N* = 3.

p62-dependent selective autophagy has been implicated as a compensatory pathway for the degradation of large ubiquitylated protein aggregates (25). However, the mechanisms that control substrate selection are poorly understood. To date, it is known that post-translational modifications on p62 determine the ability of this protein to promote selective autophagy. For instance, it has been shown that casein kinase 2 (CK2)-dependent phosphorylation of p62 at serine 403 (S403) increases the affinity of its UBA domain for polyubiquitylated chains to promote the removal of ubiquitylated substrates (42). Similarly, TANK-binding kinase 1 (TBK-1) was demonstrated to phosphorylate p62 at the same site during the infection of macrophages with *M. tuberculosis* (43). Another phosphorylation site on p62 (serine 409, S409) has also been reported to increase the affinity of its UBA domain for ubiquitylated protein aggregates. Interestingly, phosphorylation on S409 was described to be dependent on ULK1 kinase activity (25). Considering that we demonstrated that ULK1 is necessary for the ubiquitylation of bacteria and p62 recruitment, we argued whether phosphorylation on p62 S409 would also be required to recruit p62 to *L. monocytogenes* surface. For this purpose, we infected p62 knockout MEFs (p62 KO) transduced with a retrovirus for the expression of wild-type p62 (p62 WT) or its phosphorylation-null form, in which serine 409 was substituted with alanine (p62 S409A). We monitored the recruitment of p62 to the surface of *L. monocytogenes* by immunofluorescence for 2 h and observed a significant reduction in the number of bacteria associated with p62 in MEFs expressing p62 S409A when compared to MEFs expressing p62 WT (Fig. 4A through C). The quantification of the number of bacteria associated with p62 demonstrated a significant decrease in the recruitment of this protein in cells expressing the phosphorylation-null mutant when compared to MEFs expressing p62 WT (~17% in wild-type p62 vs ~1% in MEFs expressing p62 S409A, Fig. 4D). Next, we wondered if the phosphorylation on p62 S409 would also affect the recruitment of p62 by Δ*actA L. monocytogenes*. Immunofluorescence microscopy analysis of MEFs expressing p62 WT or p62 S409A did not reveal differences in the association of p62 with Δ*actA L. monocytogenes* (Fig. S9A through D). Overall, these results demonstrate that the phosphorylation on p62 S409 is crucial for efficiently recruiting p62 to the surface of wild-type *L. monocytogenes*, implying for the first time the importance of post-translational modifications in p62 in the recognition of cytosolic bacteria. Given that MEFs expressing p62 S409A displayed a significant decrease in the number of p62-positive wild-type *L. monocytogenes*, we questioned whether the lack of p62 phosphorylation at S409 would affect bacterial targeting to autophagosomes and the restriction of intracellular bacteria. To this end, GFP-LC3 p62 KO MEFs expressing p62 WT or p62 S409A were infected for 2 h with wild-type *L. monocytogenes* to quantify the number of LC3-associated bacteria. We observed that in MEFs expressing p62 WT, the number of bacteria within LC3-positive autophagosomes was significantly higher compared to MEFs expressing p62 S409A (Fig. 4E and F). Next, we wondered if the defect in targeting *L. monocytogenes* to autophagosomes would hamper the restriction of the bacteria. To test this idea, we performed gentamicin-protection assays for the quantification of viable bacteria. As demonstrated in Fig. 4G, p62 KO MEFs expressing p62 S409A present higher numbers of viable bacteria. Taken together, these data imply that in addition to the removal of protein aggregates, as previously described (25), the phosphorylation of p62 at S409 is also crucial for the restriction of bacterial replication in the cytosol.

## DISCUSSION

Xenophagy has emerged as an essential mechanism for the control of cytosolic bacteria (3). Despite significant advances in understanding the interactions between autophagy machinery and cytosolic bacteria, the mechanisms underlying the recognition of bacteria by autophagic adapters are still largely undefined. Although several studies demonstrate the importance of recruiting ubiquitin and p62 to bacterial surfaces, the factors regulating these crucial steps of xenophagy are far from elucidated. Here, we used *L. monocytogenes* as a bacterial model to exploit factors driving the recruitment of Ub and p62 during infection of non-myeloid cells. Our results demonstrate that a significant proportion of the cytosolic *L. monocytogenes* population is tagged by autophagy proteins such as LC3, Ub, and p62 in a LLO-dependent manner, in agreement with previous data (24). To understand the factors that control the ubiquitylation and p62 recruitment, we sought to investigate the role of ULK1, a major component of the ULK1 complex, known to integrate signals from energy-sensing pathways that have been previously reported to be involved in autophagy triggering during *L. monocytogenes* infection (35). Currently, the knowledge on the role of ULK1 in the autophagic response to intracellular bacteria is limited to its participation in the generation of a replicative niche for *Staphylococcus aureus* (44, 45) or *Salmonella* (46) by initiating autophagosome membrane formation. Here, we expand the role of ULK1, demonstrating that its deficiency leads to a severe impairment of bacterial ubiquitylation during xenophagy of *L. monocytogenes* that impairs the efficient handling of cytosolic bacteria. Based on previous reports demonstrating that the ULK1 complex is recruited to the bacterial surface of *Salmonella (46*), it is tempting to speculate that ULK1 might also be present on *Listeria* surface to coordinate the recruitment of ubiquitin ligases such as Parkin and Smurf1, previously implicated in the ubiquitylation of *L. monocytogenes* (10, 11).

Early reports demonstrated that ULK1 controls the phosphorylation of p62 at S409 during proteotoxic stress (25). According to the authors, ULK1-dependent phosphorylation of p62 increases the affinity of its UBA domain for ubiquitin. In light of our results demonstrating that ULK1 KO cells are limited in their ability to efficiently promote the ubiquitylation and p62 recruitment to cytosolic *Listeria*, we decided to investigate whether phosphorylation at p62 S409 would be essential for the recognition of ubiquitin on the surface of *L. monocytogenes*. Our results show that cells harboring a p62 phosphorylation null mutant in this residue display a significantly reduced capacity to localize to and target the wild-type strain of *L. monocytogenes* to autophagosomes, reducing its ability to control bacterial replication. The data presented here raise the possibility that ULK1-dependent control of xenophagy occurs at two different moments, that is, in the regulation of ubiquitylation step and p62 S409 phosphorylation. However, we must note that we cannot rule out the possibility that the reduced recruitment of p62 observed in ULK1 KO MEFs was not a mere consequence of the diminished ubiquitylation. Despite a previous report describing the TBK1-dependent phosphorylation of p62 S403 during *M. tuberculosis* infection (42), our results are the first demonstration that post-translational modifications on p62 affect the recognition of ubiquitylated bacteria and the restriction of its replication in host cytosol.

It is interesting to note that while ULK1 deficiency and blockade of p62 S409 phosphorylation deeply affected the host cell’s ability to recruit Ub and p62 during the infection with the wild-type strain, it did not impact the presence of these two proteins on the surface of Δ*actA L. monocytogenes*. It has been demonstrated that ActA mediates the escape from autophagy by mechanisms relying on actin-based motility to avoid autophagic membranes, ubiquitylation, and p62 binding to the bacterial surface (41, 47, 48). Our results raise the possibility that ActA expression might contribute to avoiding the localization of ULK1 and a putative ULK1-dependent recruitment of ubiquitin ligases to the vicinity of *L. monocytogenes*, blocking the ULK1-dependent ubiquitylation and p62 S409 phosphorylation.

Finally, our experiments demonstrating that both ULK1 deficiency and blockade of p62 S409 phosphorylation lead to impaired control of bacterial replication, together with previous work showing that the small-molecule autophagy inducer A771726 activates the AMPK-ULK1 axis to restrict the intracellular growth of *Salmonella* (49) reinforce the importance of our data and highlight the potential of ULK1 as a target for future therapeutic approaches to fight infection with intracellular bacteria.

## MATERIALS AND METHODS

### Antibodies and reagents

anti-p62 (ab109012) and anti-*L*. *monocytogenes* (ab35132) were from Abcam (Cambridge, UK). Anti-ubiquitin (ADI-SPA-203-F) was from Enzo Life Sciences (Farmingdale, NY, USA). anti-LAMP1 (sc-20011) and anti-mTOR1 (2983) were from Santa Cruz Biotechnology (Dallas, TX, USA) and Cell Signaling (Danvers, MA, USA), respectively. anti-LC3 was from Cell Signaling. Alexa Fluor 488 goat anti-mouse IgG, Alexa Fluor 488 goat anti-rat IgG, Alexa Fluor 568 goat anti-rabbit IgG, and Phalloidin Alexa Fluor 546 were from Invitrogen (Waltham, MA, USA). Anti-FLAG, Puromycin, and DAPI were from Sigma-Aldrich (Saint Louis, MO, USA). Prolong Gold Antifade reagent was from Invitrogen.

### Bacterial strains and cell culture

Wild-type *L. monocytogenes* 10403S, Δ*hly L. monocytogenes* 10403S, and Δ*actA L. monocytogenes* 10403S were kindly donated by Dr. Daniel Portnoy and Dr. Gabriel Mitchell. (Department of Molecular and Cell Biology, University of California, Berkeley, CA, USA). All bacterial strains were grown in brain-heart infusion (BHI) broth from Kasvi (Pinhais, Brazil). p62 KO MEFs retrovirally transduced with FLAG-p62 wild-type or FLAG-p62 S409A were a gift from Dr. Zhenyu Yue (Department of Neurology and Neuroscience, Friedman Brain Institute, Icahn School of Medicine at Mount Sinai, New York, NY, USA). Wild-type and ULK1 KO MEFs were donated by Dr. Douglas Green (Department of Immunology, Comprehensive Cancer Center, St. Jude Graduate School of Biomedical Sciences). Expression of GFP-LC3 in MEFs was performed as previously described (50). All cells were cultured in Dulbecco’s modified Eagle medium supplemented with 10% fetal calf serum (Invitrogen), 2 mM l-glutamine, 50 IU penicillin/50 mg/mL streptomycin (Invitrogen), and plasmocin (Invivogen, Toulouse, France). p62 KO MEFs transduced with retrovirus for FLAG-p62 wild-type and FLAG-p62 S409A were kept in puromycin (3 µg/mL). The cells were maintained in 95% air, 5% CO_2_ at 37°C.

### Bacterial infections

MEFs were seeded in 24-well plates containing glass coverslips at a density of 1 × 10^5^ cells/well in antibiotic-free medium. *L. monocytogenes* strains grown to exponential phase were centrifuged at 2,000 × *g* and adjusted to infect MEFs from wild-type and ULK1 KO mice at an MOI of 100 (p62 KO MEFs transduced with wild-type p62 or p62 S409A were infected at an MOI of 200) for 1 h in 95% air, 5% CO_2_ at 37°C. After this period, the cells were washed two times with FSC-free medium and treated with gentamicin-containing complete medium (50 µg/mL) for the indicated time points.

### Gentamicin protection assay

MEFs were infected as described above, and at the time points described in the figure legends, the cells were lysed with 0.1% Triton X-100 in PBS (PBS-T) for the quantification of viable bacteria after serial dilution of cell lysates in BHI agar plates (50).

### Immunofluorescence staining

After infection, with 4% (wt/vol) paraformaldehyde for 15 min at room temperature, blocked with PBS-BSA (1% wt/vol), permeabilized with Triton X-100 (Sigma-Aldrich), and incubated with primary antibodies for 2 h. The cells were washed three times with PBS and stained with appropriate secondary antibodies and DAPI. Coverslips were mounted with Prolong Gold.

### Image acquisition and analysis

The association of GFP-LC3, Ub, and p62 with *L. monocytogenes* was quantified from confocal images acquired with a Cell Observer Yokogawa spinning disk microscope (Zeiss, Rostock, Germany) at the microscopy facility in the Institute of Biophysics Carlos Chagas Filho (Plataforma de Microscopia Óptica de Luz Gustavo de Oliveira Castro – PLAMOL). The number of associations was quantified using ImageJ software (Schneider 2012). Plot profiles were generated using Zeiss’s Zen Desk software. Colocalization between mTOR and Lamp1 was quantified on ImageJ, using the color threshold tool to determine the colocalization area over the cell’s total area.

### Phagosomal escape assay

*L. monocytogenes*-infected cells were stained with DAPI (1:5,000 dilution) for 20 min, followed by three washes with PBS. Next, the cells were stained with Phalloidin Alexa Fluor 546 (1:500 dilution) for 60 min, followed by three washes with PBS-T. Coverslips were mounted with Prolong Gold, as described above. Bacterial escape was quantified by the number of actin-associated (phalloidin-positive) fluorescent bacteria over the total number of bacteria at 60, 120, and 240 min post-infection.

### Statistical analysis

Results are expressed as mean ± SEM of data obtained in independent experiments. Statistical differences between groups were determined using Student’s *t*-test or one-way ANOVA, Kruskal-Wallis test, with statistical significance set at *P* < 0.05.

## SUPPLEMENTAL MATERIAL

Figure S1 msphere.00308-25-s0001.tiff LC3-positive compartments surrounding *L. monocytogenes* are decorated with p62.

Figure S2 msphere.00308-25-s0002.tiff Intracellular ubiquitylation of *L. monocytogenes* is dependent on listeriolysin O.

Figure S3 msphere.00308-25-s0003.tiff *L. monocytogenes* infection reduces the colocalization of Lamp1 and mTOR.

Figure S4 msphere.00308-25-s0004.tiff *L. monocytogenes* escapes at equal rates from phagosomes of wild-type and ULK1-knockout MEFs.

Figure S5 msphere.00308-25-s0005.tiff ULK1 is required to efficiently recruit ubiquitin and p62 to the surface of *L. monocytogenes*.

Figure S6 msphere.00308-25-s0006.tiff Δ*actA* mutants are efficiently ubiquitylated and targeted by p62.

Figure S7 msphere.00308-25-s0007.tiff Ubiquitin and p62 recruitment to *L. monocytogenes* lacking *actA* do not require ULK1.

Figure S8 msphere.00308-25-s0008.tiff Ubiquitin and p62 recruitment to Δ*actA L. monocytogenes* surface do not require ULK1 at later time points.

Figure S9 msphere.00308-25-s0009.tiff Phosphorylation on p62 S409 is not required to recruit p62 to *L. monocytogenes* lacking *actA*.

ASM does not own the copyrights to Supplemental Material that may be linked to, or accessed through, an article. The authors have granted ASM a non-exclusive, world-wide license to publish the Supplemental Material files. Please contact the corresponding author directly for reuse.
