# Supplementary figures and images for: Unc-51-like kinase 1 (ULK1) regulates bacterial ubiquitylation and p62 recruitment during xenophagic clearance of Listeria monocytogenes
Source: mSphere. 2025 Aug 25;10(9):e00308-25. doi: 10.1128/msphere.00308-25 (PMC12482158; doi:10.1128/msphere.00308-25)

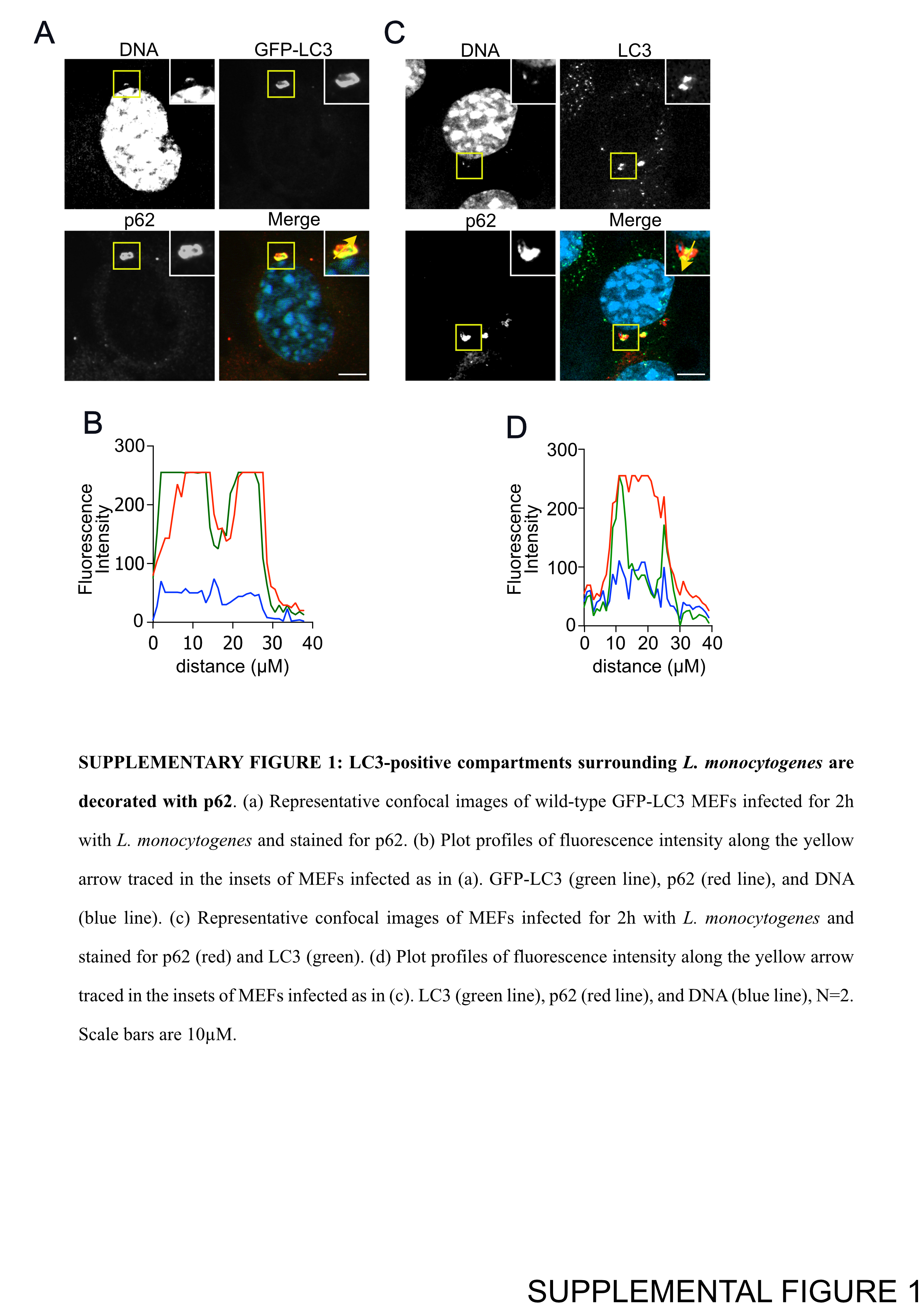

Supplement: Figure S1 — LC3-positive compartments surrounding L. monocytogenes are decorated with p62. [file msphere.00308-25-s0001.tiff]

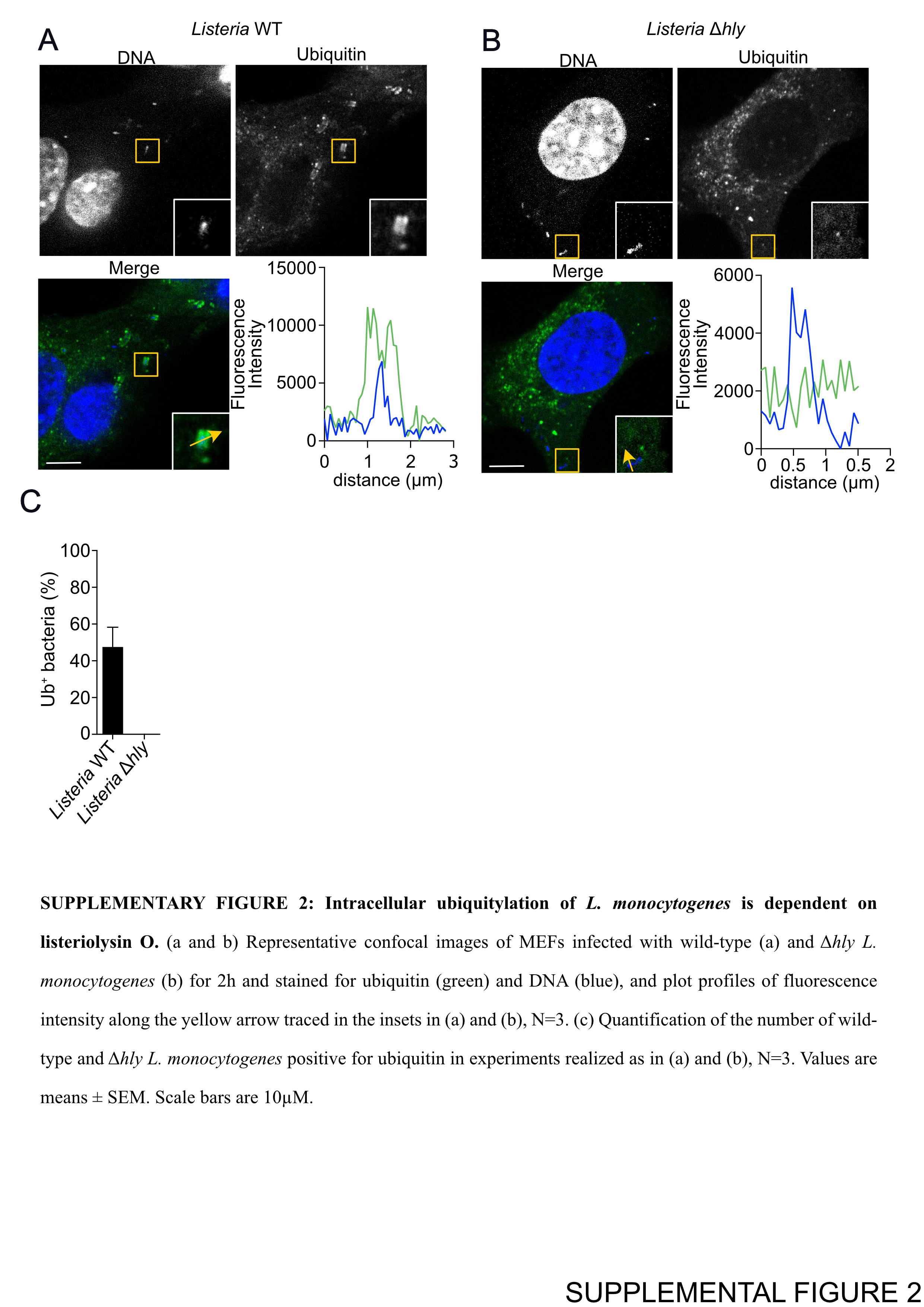

Supplement: Figure S2 — Intracellular ubiquitylation of L. monocytogenes is dependent on listeriolysin O. [file msphere.00308-25-s0002.tiff]

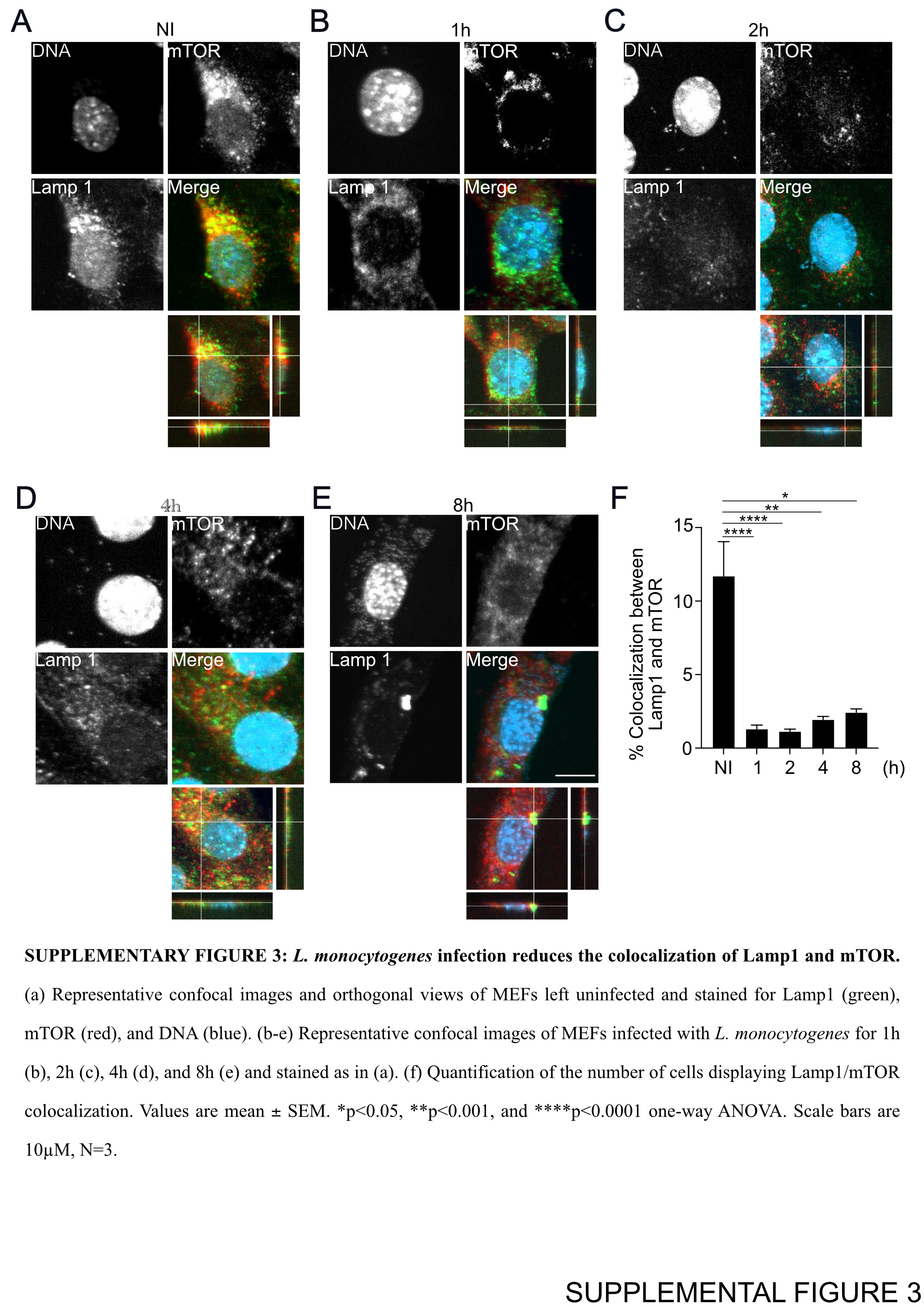

Supplement: Figure S3 — L. monocytogenes infection reduces the colocalization of Lamp1 and mTOR. [file msphere.00308-25-s0003.tiff]

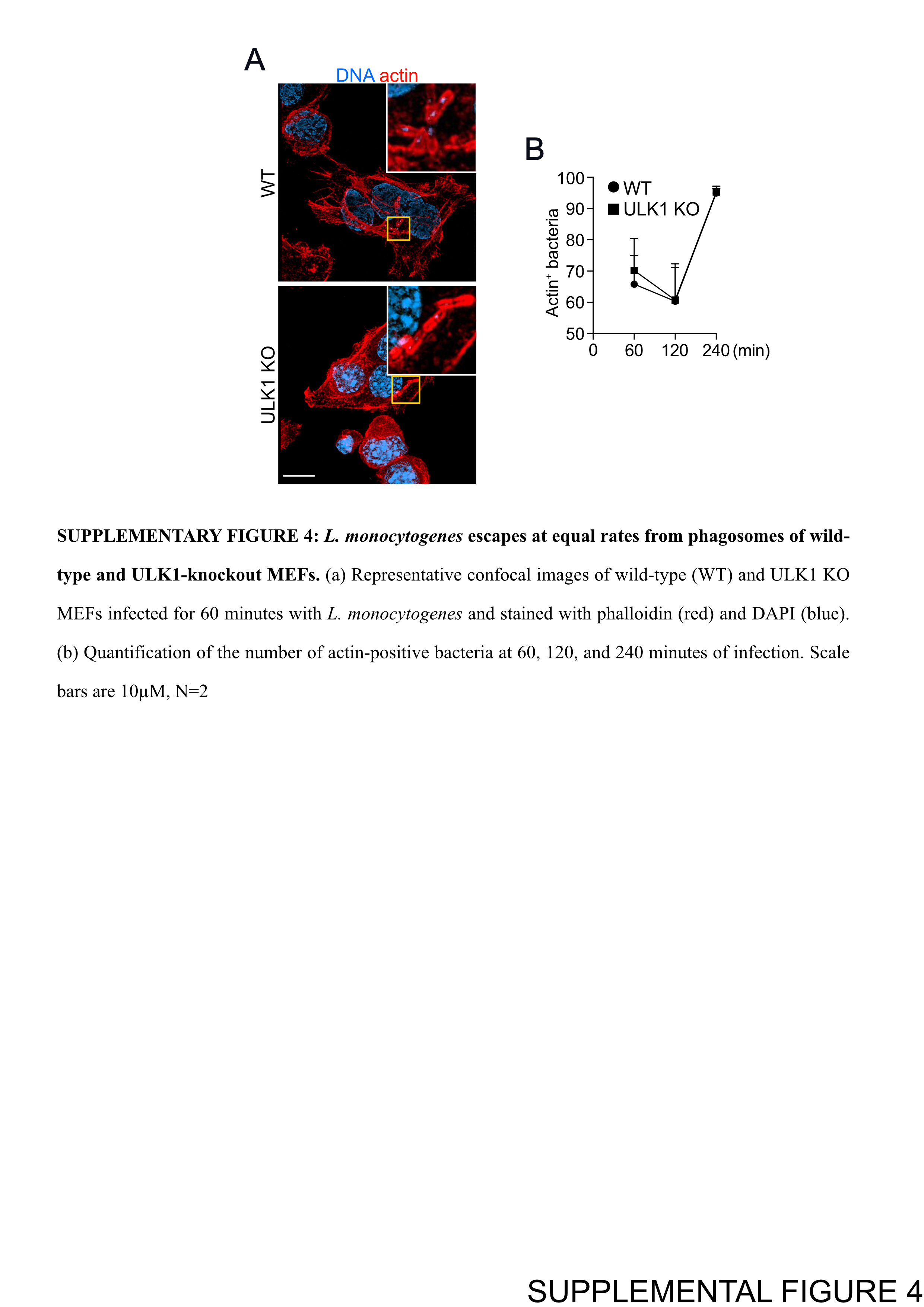

Supplement: Figure S4 — L. monocytogenes escapes at equal rates from phagosomes of wild-type and ULK1-knockout MEFs. [file msphere.00308-25-s0004.tiff]

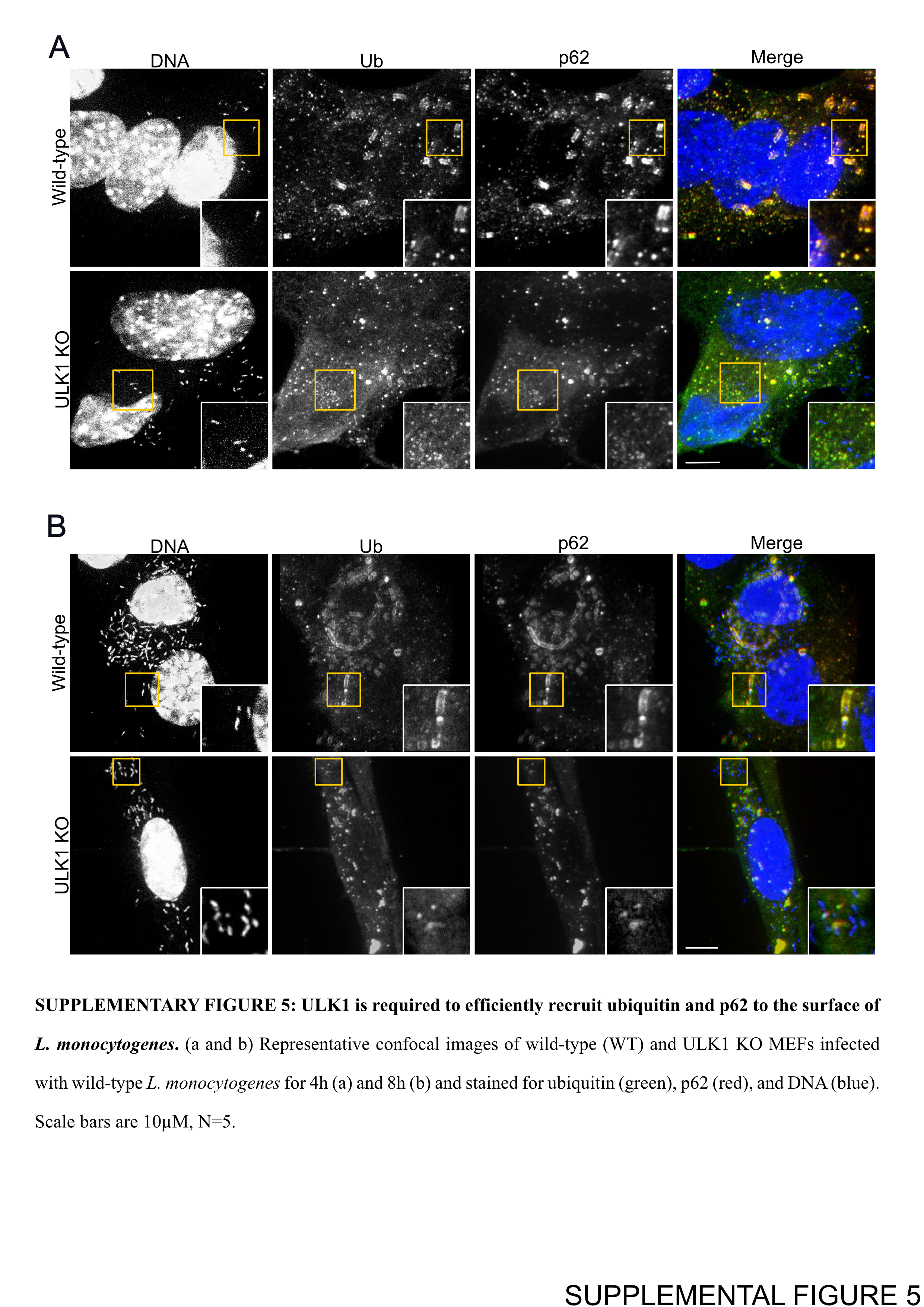

Supplement: Figure S5 — ULK1 is required to efficiently recruit ubiquitin and p62 to the surface of L. monocytogenes. [file msphere.00308-25-s0005.tiff]

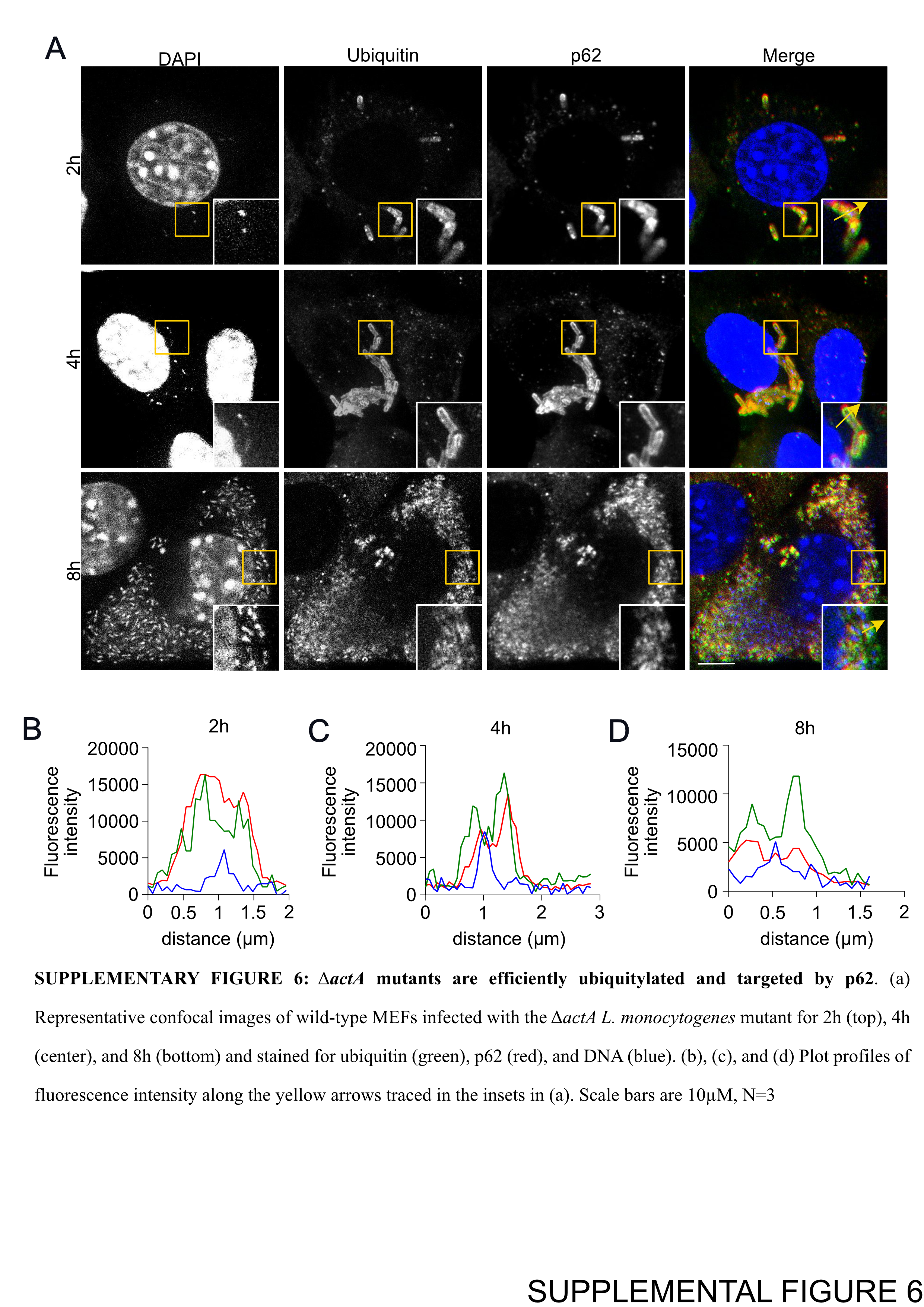

Supplement: Figure S6 — ΔactA mutants are efficiently ubiquitylated and targeted by p62. [file msphere.00308-25-s0006.tiff]

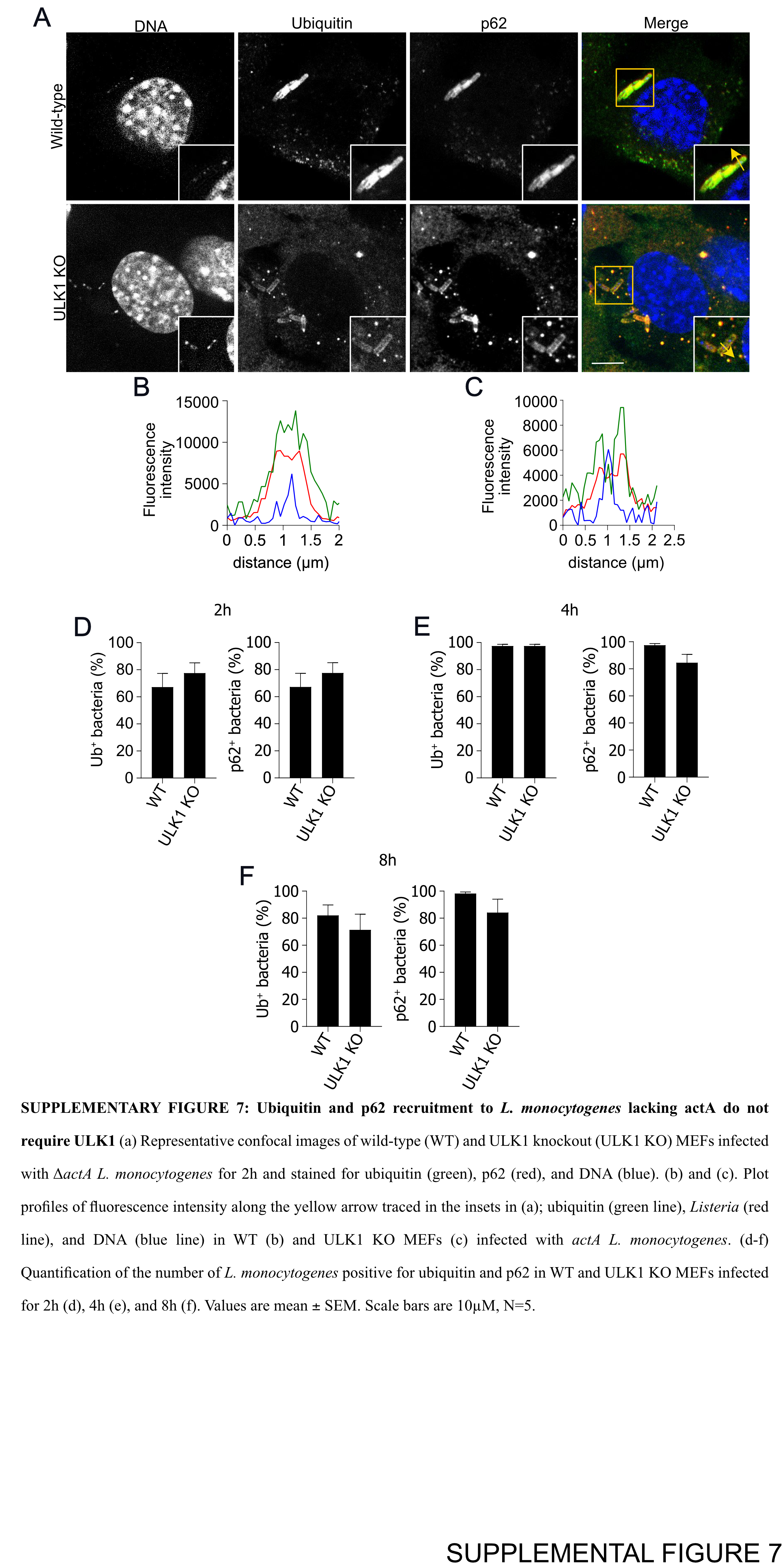

Supplement: Figure S7 — Ubiquitin and p62 recruitment to L. monocytogenes lacking actA do not require ULK1. [file msphere.00308-25-s0007.tiff]

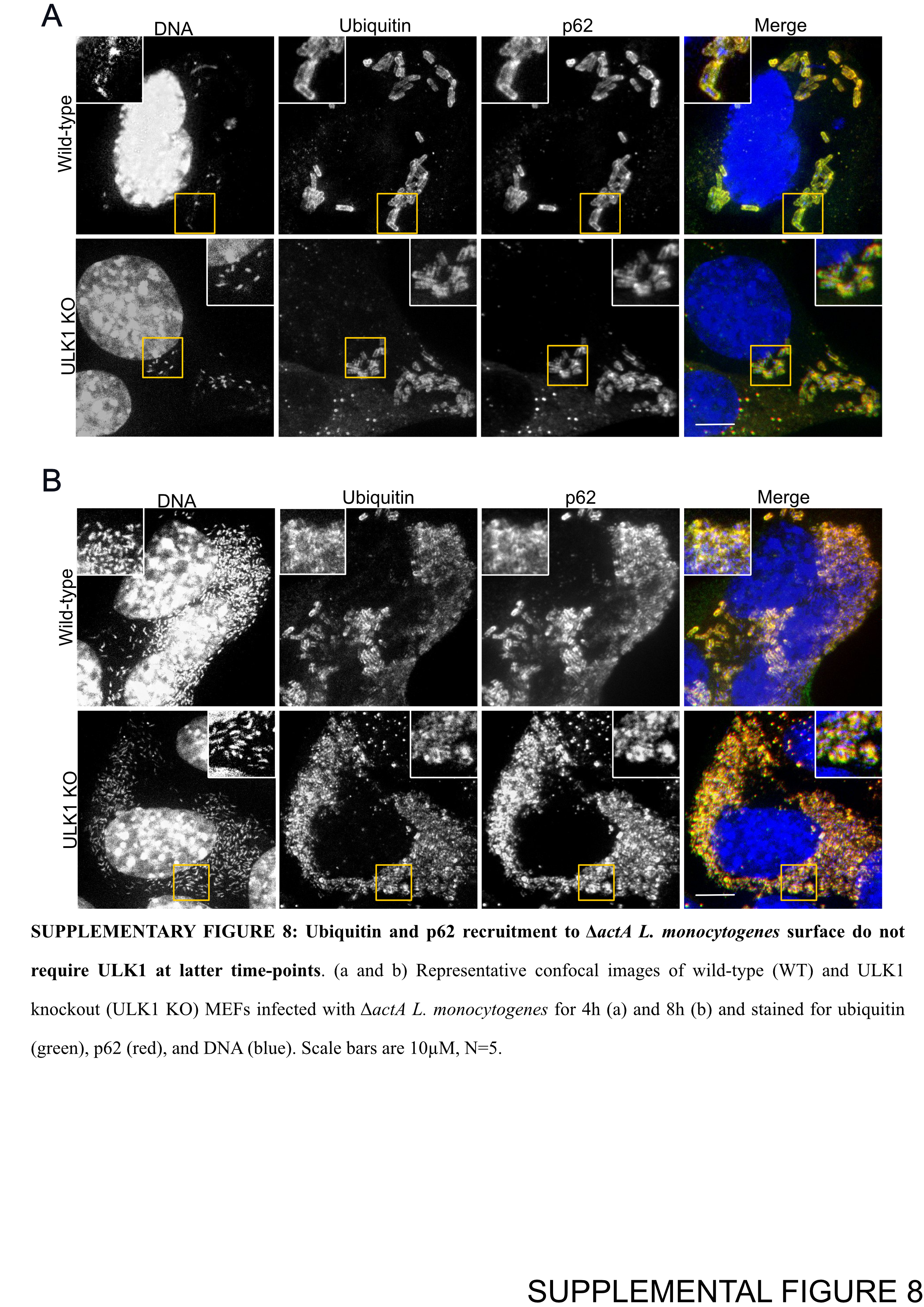

Supplement: Figure S8 — Ubiquitin and p62 recruitment to ΔactA L. monocytogenes surface do not require ULK1 at later time points. [file msphere.00308-25-s0008.tiff]

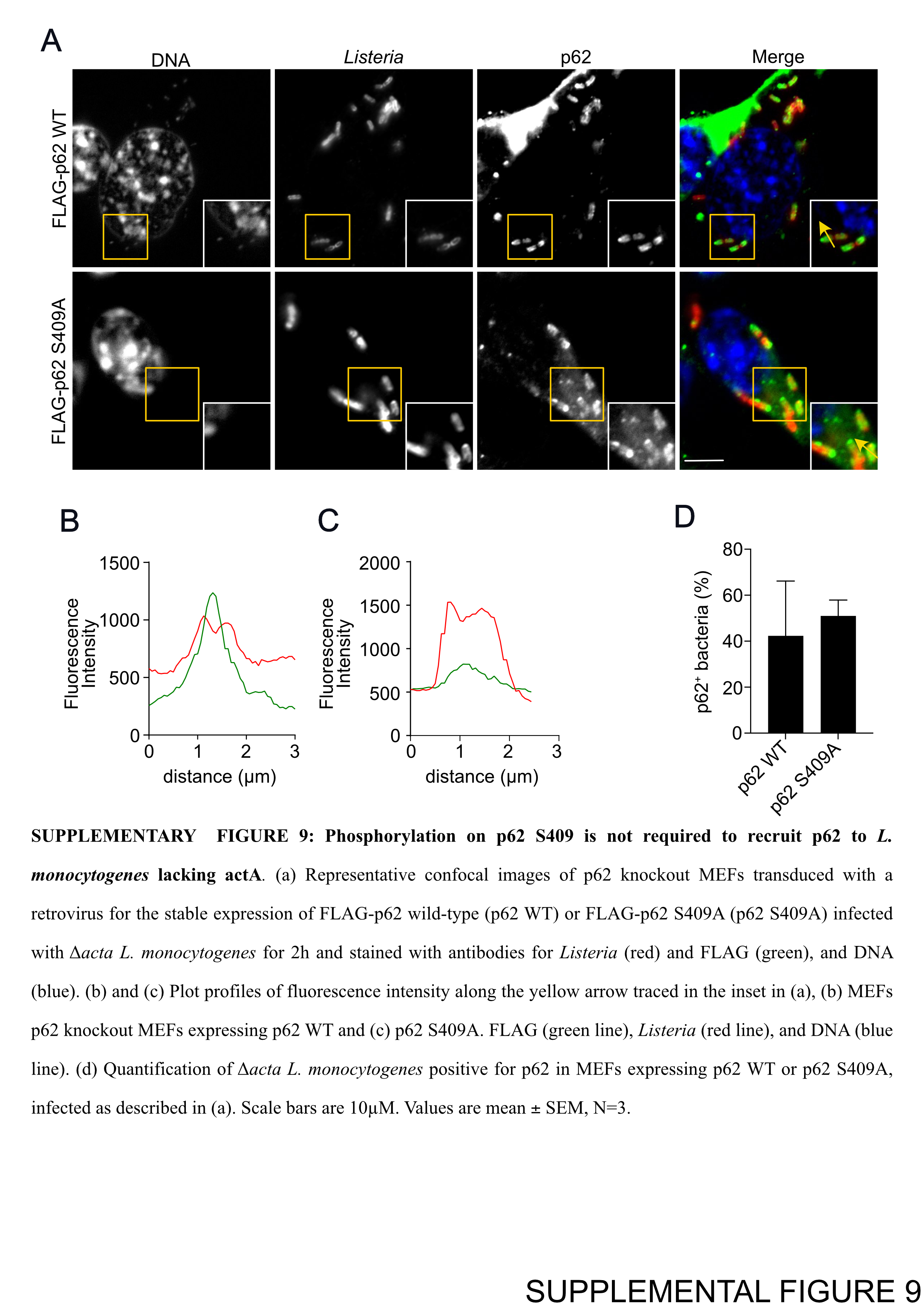

Supplement: Figure S9 — Phosphorylation on p62 S409 is not required to recruit p62 to L. monocytogenes lacking actA. [file msphere.00308-25-s0009.tiff]
